# Smart City Infrastructure Monitoring with a Hybrid Vision Transformer for Micro-Crack Detection

**DOI:** 10.3390/s25165079

**Published:** 2025-08-15

**Authors:** Rashid Nasimov, Young Im Cho

**Affiliations:** Department of Computer Engineering, Gachon University, Sujeong-gu, Seongnam-si 13120, Republic of Korea; rashid.nasimov@tsue.uz

**Keywords:** structural health monitoring, smart cities, micro-crack detection, infrastructure safety, urban infrastructure

## Abstract

Innovative and reliable structural health monitoring (SHM) is indispensable for ensuring the safety, dependability, and longevity of urban infrastructure. However, conventional methods lack full efficiency, remain labor-intensive, and are susceptible to errors, particularly in detecting subtle structural anomalies such as micro-cracks. To address this issue, this study proposes a novel deep-learning framework based on a modified Detection Transformer (DETR) architecture. The framework is enhanced by integrating a Vision Transformer (ViT) backbone and a specially designed Local Feature Extractor (LFE) module. The proposed ViT-based DETR model leverages ViT’s capability to capture global contextual information through its self-attention mechanism. The introduced LFE module significantly enhances the extraction and clarification of complex local spatial features in images. The LFE employs convolutional layers with residual connections and non-linear activations, facilitating efficient gradient propagation and reliable identification of micro-level defects. Thorough experimental validation conducted on the benchmark SDNET2018 dataset and a custom dataset of damaged bridge images demonstrates that the proposed Vision-Local Feature Detector (ViLFD) model outperforms existing approaches, including DETR variants and YOLO-based models (versions 5–9), thereby establishing a new state-of-the-art performance. The proposed model achieves superior accuracy (95.0%), precision (0.94), recall (0.93), F1-score (0.93), and mean Average Precision (mAP@0.5 = 0.89), confirming its capability to accurately and reliably detect subtle structural defects. The introduced architecture represents a significant advancement toward automated, precise, and reliable SHM solutions applicable in complex urban environments.

## 1. Introduction

Urban infrastructure integrity and safety remain a top global agenda, as they are directly related to public safety, economic growth, and the sustainable development of modern cities [1]. The gradual deterioration of infrastructure, along with rapid urban growth and more frequent extreme weather, has significantly increased the demand for structural health monitoring (SHM) methods that are both reliable and proactive [2]. The dominant approaches to structural assessment, visual inspections, and manual interventions are very labor-intensive, expensive, subjective, and prone to errors, especially when it comes to detecting subtle and fine-grained anomalies like micro-cracks, corrosion, delamination, and the beginning stage of structural degradation [3]. These drawbacks are revealed in the fact that the need for more efficient, precise, and even automated inspection methods, which are capable of finding early defects, helping with the administration of interventions, and continuously monitoring infrastructure conditions, is becoming more and more urgent [4].

The latest artificial intelligence (AI) technologies, such as deep learning and computer vision, offer a new approach in solving different SHM problems [5]. Convolutional Neural Networks (CNNs) are the main frameworks that have always been used for automated defect detection, and they demonstrate strong performance in extracting hierarchical spatial features from image data related to structural deterioration [6]. Nevertheless, CNN-based approaches have various restrictions that affect their ability to provide full local reasoning for global context, mainly because they are based on local receptive fields and have a spatial limitation that is typical of convolving operations. Consequently, they become less sensitive to small or fine defects such as micro-cracks and structural irregularities [7].

Transformers (ViTs) have become a hot topic in computer vision as structures that can do away with CNN architectures due to their superior capability to identify similarities and patterns across large spatial regions, thus enabling powerful anomaly detection and localization [8]. Unleashing a self-attention mechanism, ViTs have proven to be able to efficiently cover very large receptive field areas—a known shortcoming of traditional CNNs—and have recorded excellent performance in several computer vision tasks, including image classification, segmentation, and object detection [9]. On the downside of these advantages, the use of ViTs in the structural health monitoring (SHM) arena is still difficult, especially in catching intricate local structural information, which is a vital requirement for the detection of subtle anomalies [10]. The source of this limitation lies in the self-attention mechanism, which mostly concentrates on the global context; consequently, local features become less emphasized [11].

As an attempt at solving these issues, this paper suggests an AI-based, innovative, and extremely efficient SHM regime that combines the potentials of ViT-based architectures with a freshly created Local Feature Extractor (LFE) unit, incorporated in the modified DETR framework. The most important part of the ViT-based DETR model that is presented is by far the LFE component, which is conspicuously more capable of extracting complex local features, thus greatly improving the model’s capability of detecting minor structural defects, the ones that deep learning models usually cannot see. The LFE component is made up of several convolutional layers with residual connections added and non-linear activation functions, thus continuously refining the feature and the stability of the gradient being propagated. This architecture not only enables local and global information to be fused seamlessly, but it also results in quite a big increase in the detection accuracy, precision, recall, and overall generalization capability.

To confirm the proposed method, comprehensive experimental evaluations have been carried out utilizing the publicly available benchmark dataset SDNET2018 as well as a custom dataset consisting of high-resolution images of damaged bridge components that were collected from real urban environments. These datasets represent an extensive variety of structural conditions, deterioration patterns, and environmental factors, thus offering a solid and realistic testing ground. Several state-of-the-art deep learning detection architectures, such as DETR variants and multiple YOLO (You Only Look Once) versions (YOLOv5 to YOLOv9), were used for comparative analyses to demonstrate that the proposed ViLFD model is the best among all the models in terms of accuracy and robustness. The results show that the proposed technique brings considerable performance advances across the principal evaluation metrics such as accuracy, precision, recall, F1-score, and mean Average Precision (mAP@0.5), and thus leads amongst the current state-of-the-art techniques.

The main contributions of this paper are summarized as follows. First, we propose an innovative hybrid architecture that integrates a Vision Transformer backbone with a newly designed Local Feature Extractor (LFE) module to enhance sensitivity and accuracy in detecting small-scale structural anomalies. Second, we conduct extensive experiments using two datasets—one benchmark and one custom-built—to demonstrate the robustness and practical applicability of the proposed framework under diverse urban infrastructure conditions. Finally, our empirical analysis, supported by rigorous comparative studies, highlights the significant performance gains achieved by our model, marking a notable advancement in the development of automated structural health monitoring techniques. The remainder of this paper is organized as follows. Section 2 presents a detailed review of related work, covering recent advancements and existing challenges. Section 3 describes the proposed model architecture, including the technical rationale behind each component. Section 4 outlines the experimental setup and datasets used, followed by results and comparative analysis. Section 5 discusses the key findings and their implications for structural health monitoring. 

## 2. Related Works

Advanced deep learning techniques for structural health monitoring (SHM)—such as automated crack and anomaly detection in road infrastructure—have gained significant popularity due to the limitations of traditional visual inspection methods [12]. This paper presents a comprehensive overview of computer-vision-based approaches, including CNN-based, transformer-based, and hybrid methods. It outlines the current state of the field and identifies several challenges that form the core focus of our research. In the past, structural defect detection has mainly depended on computer vision techniques that are conventional, like threshold-based segmentation [13], edge detection algorithms [14], wavelet transforms [15], and feature extraction methods that are handcrafted [16]. To give an example, edge detection techniques like the Canny and Sobel operators were among the most widely used for identifying visible cracks in concrete and asphalt surfaces [17]. However, these methods are very sensitive to noise coming from the environment, lighting changes, and surface irregularities that drastically reduce their accuracy and reliability [18]. Not only that, but these feature-based approaches also require manual parameter tuning that is very extensive, and they lack generalizability most of the time, which makes them very hard to apply in complex real-world scenarios [19].

On the other hand, the use of CNNs has led to a significant improvement in feature extraction and hierarchical representation learning, allowing for a new era of automated defect detection. The first CNN-based trial in the field of concrete crack detection [20] showed that simple CNN architectures can perform much better than traditional techniques. Later developments introduced deeper architectures like ResNet, DenseNet, and InceptionNet, which brought significant increases in classification and segmentation accuracy for different environmental conditions [21]. Let us cite an example: the DeepLabV3+ model, a deep learning model for crack detection by [22], opened up the possibilities of addressing changes in image scale and increasing the spatial resolution of atrous convolutions, thus leading to performance gains. In addition to these breakthroughs, CNNs have an intrinsic limitation owing to the fixed receptive fields and local convolutional operations that prevent them from grasping long-range dependencies and global context, which are essential in finding very subtle or small defects [23]. Alternatives such as Vision Transformers (ViTs) have been launched recently as strong competitors, providing more effective global context understanding via self-attention methods [24]. A major milestone was the introduction of the DETR by Carion et al. [25], which replaced traditional anchor-based object detection pipelines with a novel end-to-end transformer-based set prediction framework. The adoption of transformer-based models has facilitated promising progress in structural inspection tasks, including crack localization, anomaly detection, and defect segmentation [26]. However, it has been argued that ViT-based models alone face challenges in capturing local patterns essential for micro-defect detection—an important limitation in structural health monitoring (SHM) applications [27].

To address these challenges, hybrid architectures that combine CNN and ViT modules have gained popularity. These models aim to leverage both local spatial feature extraction and global context modeling. For example, ref. [28] proposed a CNN–transformer hybrid model for surface defect detection, achieving improved accuracy by integrating convolutional layers with transformer-based attention mechanisms. Similarly, ref. [29] introduced hybrid deep learning frameworks that combine local convolutional operations with transformer modules for global reasoning, demonstrating strong performance in detecting subtle surface anomalies in complex inspection scenarios. However, most current hybrid models rely on relatively simplistic integration strategies—such as feature concatenation or attention-weighted summation—that fail to fully exploit the synergistic potential of combining local and global features [30]. Furthermore, existing models generally do not explicitly address the detection of subtle, fine-grained defects such as micro-cracks with sufficient robustness and precision. Only a limited number of models incorporate specialized local feature enhancement mechanisms tailored to infrastructure inspection. As a result, their effectiveness diminishes when generalizing to visually complex, noisy, and environmentally variable real-world scenarios [31]. Motivated by these unresolved issues, this paper proposes a novel hybrid deep learning framework for the structural health monitoring of urban infrastructure, specifically designed to identify fine-grained structural defects. Our approach employs a modified DETR architecture with a ViT backbone enhanced by a purpose-built Local Feature Extractor (LFE) module. This architectural innovation effectively addresses the limitations of prior models by significantly improving the capacity for detailed local feature extraction, which is critical for detecting micro-level anomalies. The proposed model preserves the global reasoning capabilities of ViT-based attention while enhancing local spatial representation through the newly designed LFE block. Extensive experimental evaluations—using both benchmark and self-collected infrastructure datasets—demonstrate that our model outperforms existing state-of-the-art methods by a significant margin. The following sections provide a comprehensive description of the proposed architecture, experimental methodology, and results, along with a detailed analysis of the findings and their implications for deep learning-based SHM systems.

## 3. Materials and Methods

This paper describes a deep learning framework with a modified DETR architecture for the structural health monitoring of urban infrastructure. The proposed method incorporates a ViT as the backbone of the baseline model, substituting the original CNN-based feature extractor. To further increase the model’s capability to detect small defects, we propose a new module called the Local Feature Extractor (LFE). The LFE module is designed to capture detailed local features, thus increasing the sensitivity of the proposed model to minor defects like micro-cracks. This architectural improvement not only greatly enhances the detection accuracy and the robustness in scarred urban environments but also allows the detection system to work with a wide variety of sensors.

### The Proposed Model

**Figure 1:**
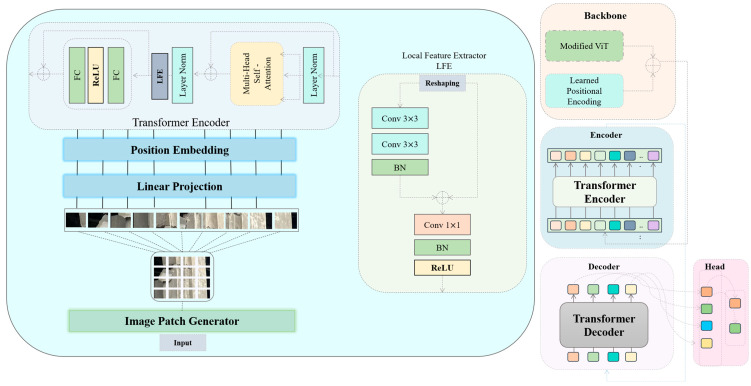
The architecture of the proposed ViT+LFE-DETR model. The ViT backbone, shown in blue, is responsible for capturing global contextual information, while the LFE block, shown in orange, enhances fine-grained local spatial features. The dashed line between them represents the data flow from the ViT output into the LFE block, ensuring that global and local features are effectively fused before the transformer encoder processes them further.

As illustrated in Figure 1, we propose a novel approach for crack detection in structural components. In this framework, the standard ViT model is modified through the integration of a custom LFE block. This block is designed to enhance the ability of the proposed model to capture fine-grained and complex local features, which are then forwarded to subsequent layers within the transformer encoder for further processing. Architecturally, the LFE block is lightweight and consists of two convolutional layers: the first employs 3 × 3 kernels to extract spatial features, while the second utilizes a 1 × 1 kernel to refine and adjust channel dimensions, enabling efficient integration with the transformer pipeline:

(1) $$F_{TE}=\;MLP(F_{LFE}(F_{LN}(MSA(F_{LN}(F_{PE}))+F_{PE})))$$

Equation (1) outlines the complete computational flow within the transformer encoder, $F_{TE}$. Initially, the input feature map $F_{PE}$, augmented with positional embeddings, undergoes Layer Normalization (*LN*) $F_{LN}$ to standardize the input distribution, a necessary step prior to the Multi-Head Self-Attention (MSA) mechanism, ensuring compatibility in feature scale and dimensionality. Following the MSA operation, an element-wise addition is performed between the input features and the MSA output to preserve the original information and mitigate feature degradation. Subsequently, a second LN is applied, after which our proposed LFE block is introduced, as mathematically formalized in Equation (2):

(2) $$F_{LFE}=F_{1}=BN(F_{3\times 3}(F_{3\times 3}(F_{LN\_out})))\;+\;F_{LN\_out},\;F_{2}=\;max(0,\;BN(F_{1\times 1}(F_{1})))$$

Before applying the 3 × 3 convolution in the LFE block, the transformer encoder output—initially a sequence of flattened patch embeddings of dimension *N* × *D*, where *N* is the total number of patches and *D* is the embedding dimension—is reshaped back into a spatial grid. Specifically, the sequence is reorganized into a feature map of size *H*_*p*_ × *W*_*p*_ × *D*, where *H*_*p*_ and *W*_*p*_ denote the patch grid dimensions along the height and width, respectively. This reshaping step restores the spatial arrangement of patches to their corresponding positions in the original feature map, thereby preserving local neighborhood relationships. Standard zero-padding is applied during convolution to ensure that spatial alignment is maintained. As a result, the subsequent 3 × 3 convolution can effectively capture fine-grained local context while retaining the positional information provided by the transformer’s positional embeddings. The LFE block is architecturally segmented into two sub-modules, denoted as *F*_1_ and *F*_2_. The first sub-module, *F*_1_, processes the output of the preceding Layer Normalization as its input and applies two sequential convolutional layers. These layers are specifically designed to extract complex and fine-grained features, $F_{3\times 3}$, thereby enhancing the sensitivity of the proposed model to minute structural anomalies such as micro-cracks. To stabilize training and mitigate the vanishing gradient problem, a residual connection is employed between the input feature map and the output of this sub-module. This not only reinforces gradient flow but also aids in the preservation and restoration of critical semantic information. The second sub-module, *F*_2_, focuses on the channel-wise adjustment of the extracted features. It employs a 1 × 1 convolution to align the channel dimensions $F_{1\times 1}$, followed by the application of the ReLU activation function to introduce non-linearity and improve feature expressiveness. Together, these components enable the LFE block to effectively bridge local feature enhancement and global attention, contributing to more robust crack detection performance. Table 1 presents the full architecture of the LFE block.

The proposed ViT+LFE-DETR model employs a patch embedding dimension *D* = 768 for the ViT backbone, with each image divided into patches of size 16 × 16 pixels. The transformer encoder consists of 12 layers, each with 12 attention heads. The LFE block processes feature maps with an input channel size matching *D* and applies a 3 × 3 convolution followed by another 3 × 3 convolution with identical stride and padding settings, both preserving spatial resolution. The channel adjustment stage uses a 1 × 1 convolution to refine dimensions before integration into the transformer pipeline. Table 1 summarizes the dimensions and hyperparameters of each major stage. To train the proposed crack detection model effectively, we adopt a composite loss function that balances object localization, classification, and boundary accuracy, Equation (3):

(3) $$L_{total}={\lambda_{cls}L}_{cls}+{\lambda_{loc}L}_{loc}{{\;+\lambda }_{mask}L}_{mask}$$

where $L_{cls}$ is the classification loss, $L_{loc}$ is the bounding box regression loss (localization), and $L_{mask}$ is the binary segmentation loss used for precise crack boundaries. $\lambda_{cls}$, $\lambda_{loc}$, and $\lambda_{mask}$ are weighting factors empirically set to balance the influence of each component. Table 2 presents the ablation results, demonstrating the effectiveness of integrating the ViT and the proposed LFE into the DETR framework. The baseline DETR model achieves an accuracy of 92.02% with an F1-score of 0.89. Replacing the original backbone with ViT alone improves performance to 93.11% accuracy and 0.90 F1-score. Incorporating both ViT and the LFE block yields the highest accuracy of 95.0% and an F1-score of 0.93, confirming the significant performance gain contributed by the LFE in extracting finer features crucial for crack detection.

## 4. Experiments and Results

### 4.1. Dataset

**Figure 2:**
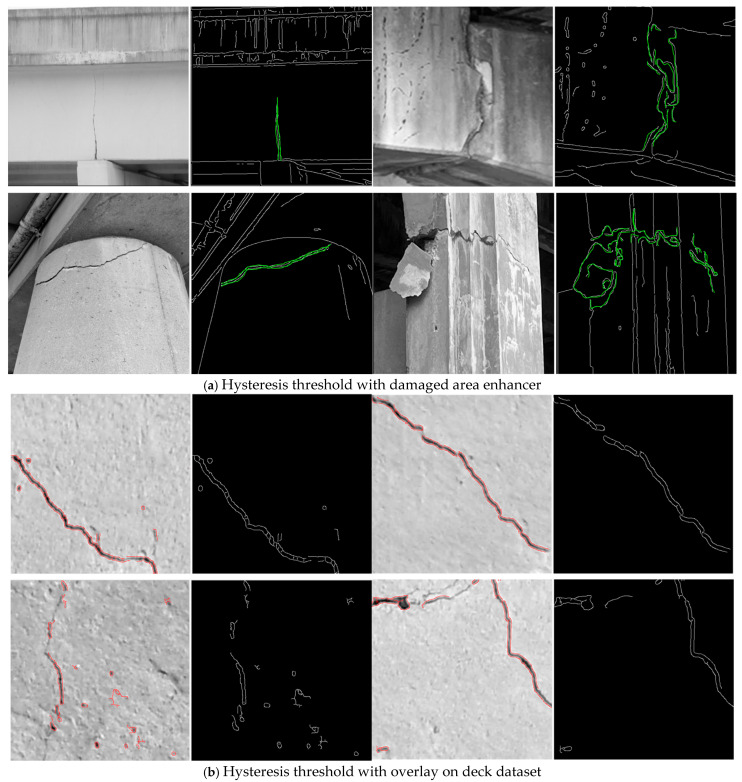

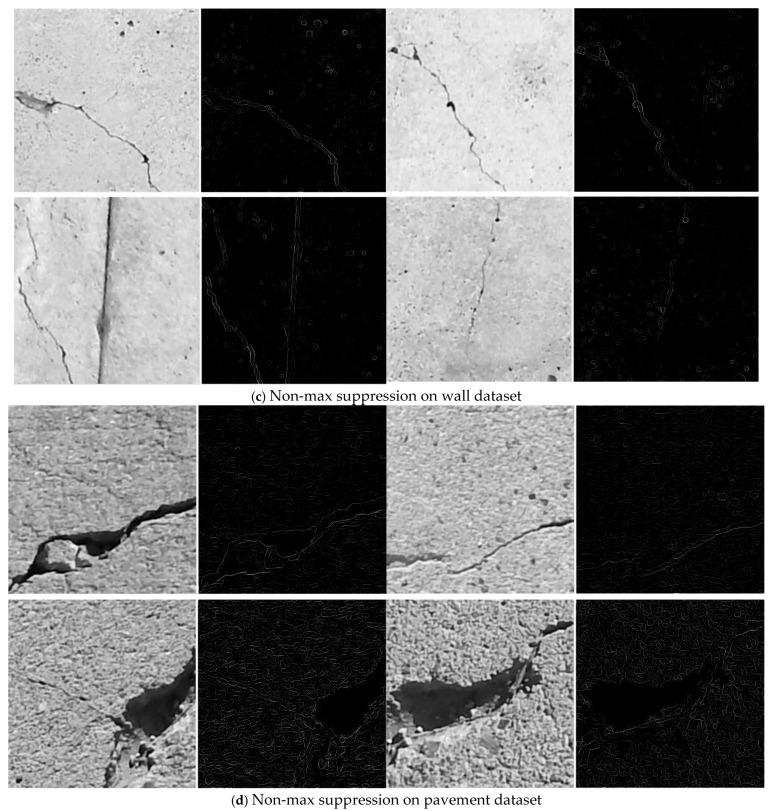
The results of the Canny edge detector on the (**a**) custom dataset, (**b**) deck dataset, (**c**) wall dataset, (**d**) pavement dataset.

To evaluate the performance and generalization capability of our proposed model, we conducted experiments on two datasets: a public benchmark dataset SDNET2018 and a custom-collected dataset comprising images of damaged bridges from real-world infrastructure. The SDNET2018 dataset is a comprehensive and widely adopted benchmark for surface crack detection, comprising over 56,000 high-resolution images of concrete structures. The images cover a variety of structural elements, including bridge decks, concrete walls, and pavements, under diverse environmental and lighting conditions. Each image, sized at 256 × 256 pixels, is labeled with binary annotations indicating the presence or absence of cracks. The dataset is rather challenging because it includes noise factors like surface stains, paint marks, and joint lines that shadows may make lighter, and these local features often resemble cracks. On top of that, the fact that crack width, orientation, and scale can vary considerably makes the task even more complicated. To train the models, the dataset was divided into three parts—training, validation, and test—using the ratio of 70:15:15. Data augmentation, which includes random rotations, flips, contrast changes, and geometric transformations, was employed lavishly to consolidate the model’s strength and also to prevent overfitting. Along with the SDNET2018 benchmark, we created a local dataset including the specific damaged bridge components seen in the real-world situation of structural monitoring. This local dataset is composed of 1200 RGB images with a resolution of 512 × 512 pixels which were taken by DSLR cameras and UAV-mounted imaging systems during field inspections in urban environments (Figure 2).

These images depict different types of structural degradation, such as longitudinal and transverse cracks, delamination, and spalling, in various materials, including reinforced concrete and asphalt surfaces. The dataset receives more inputs from the elements of nature, such as vegetation, shadowing, traffic lines, and also weather-induced surface variations. Through the use of the LabelMe tool, domain experts provided very good annotations of all crack regions at the pixel level. The dataset was divided into 800 training images, 200 for validation, and 200 for testing. Additionally, advanced augmentation strategies were employed to simulate domain-specific distortions such as surface weathering, motion blur, and varying illumination, enabling the model to learn more generalized features applicable to real-world inspection conditions.

### 4.2. Comparison with Baseline Models

Table 3 presents a detailed quantitative evaluation of our proposed architecture in comparison with multiple state-of-the-art object detection models, including DETR variants and the YOLO family (v5 to v9). All experiments were independently repeated five times with different random seeds to ensure statistical robustness. For each metric, the mean and standard deviation are reported (mean ± std). In addition, paired t-tests were conducted between the proposed ViT+LFE-DETR and the strongest baseline model to verify statistical significance. The results show that improvements in accuracy, precision, recall, F1-score, and mAP@0.5 are significant at *p* < 0.05, indicating that the performance gains are unlikely to be due to random variation.

**Figure 3:**
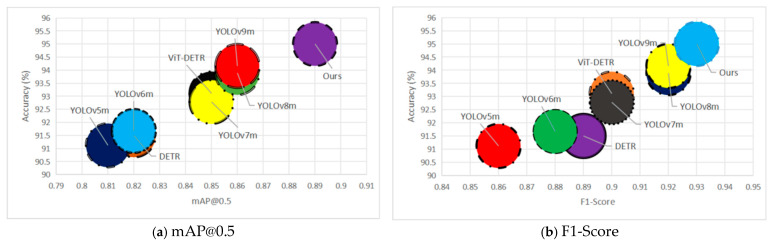

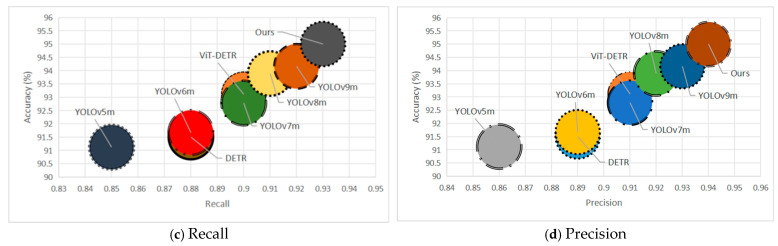
The visualization of the comparison of different metrics to the accuracy of the baseline models.

The baseline DETR model yields an accuracy of 91.5%, precision of 0.89, and F1-score of 0.89, with a mean Average Precision at an IoU threshold of 0.5 (mAP@0.5) of 0.82, indicating limited sensitivity to fine-grained structural defects. Replacing the standard CNN backbone with a ViT improves overall performance, as reflected in the ViT-DETR configuration, which achieves 93.11% accuracy and a mAP of 0.85, highlighting the contribution of global contextual encoding. The YOLO-based models exhibit incremental improvements across versions, with YOLOv9m reaching up to 94.16% accuracy and a strong F1-score of 0.92, demonstrating their robustness in general object detection tasks. However, despite their efficiency, these models are not explicitly optimized for the detection of fine, irregular patterns such as surface cracks Figure 3.

**Figure 4:**
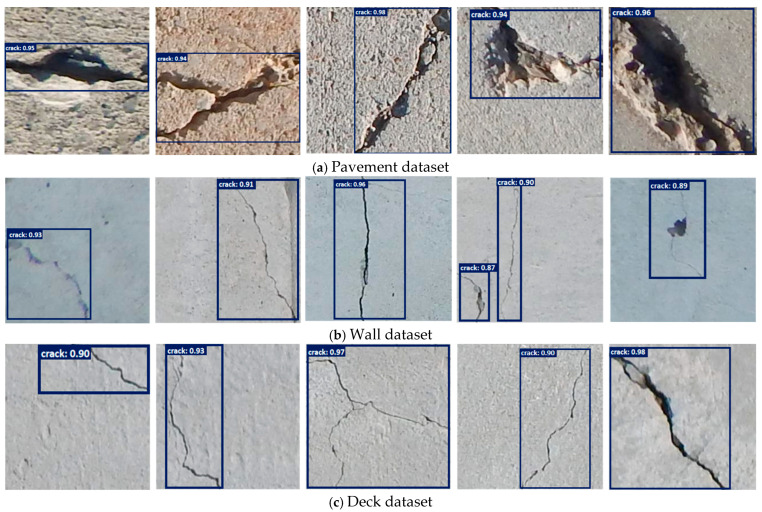
The validation results of the proposed model.

In contrast, our proposed model achieves SOTA performance across all evaluation metrics. It records the highest accuracy of 95.0%, precision of 0.94, recall of 0.93, F1-score of 0.93, and mAP@0.5 of 0.89. These results underscore the efficacy of integrating the LFE block with ViT-based attention mechanisms within the DETR framework, Figure 3. The LFE module enhances the ability of the proposed model to capture the subtle, high-frequency spatial patterns associated with micro-cracks, while the ViT backbone contributes strong global reasoning. Together, they provide a synergistic boost in both detection sensitivity and precision, affirming the superiority of our architecture for structural health monitoring applications, Figure 4.

### 4.3. Comparison with SOTA Models

To gauge our ViLFD framework’s effectiveness and generalization ability, we went to lengths to compare it with prominent state-of-the-art (SOTA) models across four main categories: traditional image processing methods, CNN-based architectures, transformer-based models, and hybrid deep learning approaches. This comparison was performed with consistent criteria using the SDNET2018 benchmark and our handmade real-world infrastructure dataset. In early SHM methods, the main reliance was on handcrafted feature extraction and image filtering techniques similar to the edge detection and wavelet transforms shown in Figure 4.

To give an example, ref. [13] employed thresholding along with multistep watershed segmentation, and Jahanshahi et al. [14] utilized Canny and Sobel operators for bridge crack detection. In addition, ref. [15] resorted to continuous wavelet transforms combined with infrared thermography, while [16] were able to detect damage automatically through low-level descriptors. These methods, although simple, interpretable, and light, are very vulnerable to noise and hardly capable of generalizing across different urban environments. CNNs have gone a long way in promoting SHM by capturing hierarchical spatial features. Ref. [20] proved that even shallow CNNs can do better than the traditional techniques; on the other hand, Abbas and Alghamdi [21] proposed models of semantic segmentation for surface cracks. DeepLabV3+ variants [22,23] gave the possibility of better scale invariance and resolution. In particular, ref. [24] worked in the direction of integrating feature pyramids with CNNs for robust edge segmentation. YOLO-based models—YOLOv5m through YOLOv9m [27]—dominate the area of real-time crack detection because of their speed and simplicity. But despite YOLOv9m reaching 94.16% accuracy and an F1-score of 0.92, it is still lacking mechanisms for subtle local features Table 4.

**Figure 5:**
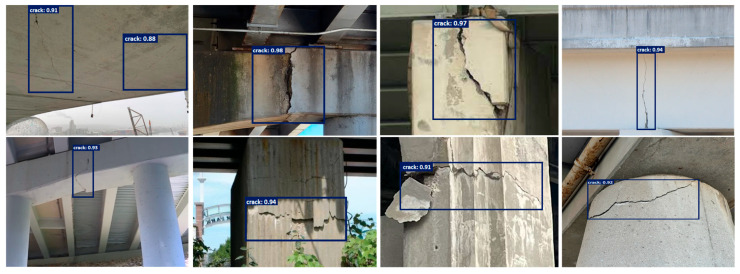
The test results of the proposed model on the custom dataset.

ViTs have completely changed the computer vision field by representing the dependencies of far-apart areas. Reference [25] presents DETR, a transformer-based end-to-end detection architecture, and [9] introduced Adaln for multi-domain learning in SHM, whereas [10] transferred transformers for social-media-informed infrastructure safety analysis. Although ViTs dominate global contextual understanding, their performance in the micro-defect detection field is, most of the time, severely limited due to the local feature weakness, as the authors of articles [11,24] reported, unlike the proposed model’s successful detection, shown in Figure 5.

To go beyond individual barriers, recent research combines CNNs with transformers. Refs. [28,29] came up with hybrid models, which connect local convolution with transformer attention. Ref. [30] presented a complementary fusion network, and at the same time, ref. [31] constructed a multi-scale transformer (ETAFHrNet) that was specifically designed for asymmetric pavement crack segmentation. Although the models got better, they generally use simple concatenation or attention-weighted summation, which means they are only partially utilizing the interplay between global and local cues. The ViLFD model mitigates the global reasoning power of the Vision Transformer with a new Local Feature Extractor (LFE) module. The LFE makes use of residual-enhanced convolutional layers, which helps it to follow high-frequency patterns that are essential for detecting micro-cracks and very small abnormalities. In contrast to previous hybrids, our design carries out explicit local improvement within the transformer encoder, thus being able to achieve considerable increases in accuracy and robustness not only under the conditions of noise but in real situations as well.

## 5. Conclusions

This paper introduces ViT+LFE-DETR, an innovative deep learning architecture that goes a long way in revolutionizing structural health monitoring (SHM) by fusing the global attention capability of ViTs with a specially designed LFE unit. The proposed architecture is optimized by experiments run on both benchmark SDNET2018 data and a custom dataset of urban bridge components. The experimental results demonstrate that the new architecture can detect slight and fine-grained defects in the infrastructure, such as micro-cracks, with superior accuracy, robustness, and generalization. The authors have performed comparative evaluations against existing SOTA models, including traditional image processing methods, CNN-based systems, pure transformer models, and hybrid architectures. These evaluations show that the proposed framework is superior, particularly in environmental noise and visually complex infrastructure surfaces. The LFE module is an important element in the model, as it not only enhances the model’s sensitivity to local features of structures but also effectively solves the issue of ViTs’ global context awareness and the spatial specificity that is still needed to accomplish SHM tasks. The discussed method explicitly enables high-frequency detail extraction and guaranteed feature propagation, and it achieves first place in multiple metrics (accuracy 95.0%, precision 0.94, recall 0.93, F1-score 0.93, mAP@0.5 0.89). The achieved results collectively indicate the feasibility of ViLFD as an efficient, scalable, and reliable solution for automated SHM applications in realistic urban settings. Planned future work will extend the model’s ability in multimodal sensor data handling, incorporating temporal dynamics for continuous damage diagnosis and establishing the framework in the entire infrastructure of a smart city monitoring system.

In future work, we aim to extend the proposed ViT+LFE-DETR framework beyond purely image-based analysis by incorporating structural context variables such as the type of structural member, material composition, and external load/support configurations. These multimodal inputs could be integrated through an auxiliary metadata encoder fused with the visual features, allowing the model to leverage both geometric and material context in its crack detection process. We anticipate that this context-aware approach will improve the model’s generalization capability and diagnostic value in real-world structural health monitoring scenarios. For reinforced and prestressed concrete members, extensive cracking patterns are frequently linked to advanced degradation processes driven by shear and torsion forces. Capturing and integrating such mechanical load information into the proposed ViT+LFE-DETR framework could enhance the interpretation of detected cracks and improve the accuracy of severity assessment. Future work will explore multimodal fusion approaches where mechanical stress parameters—obtained from structural analysis models, sensors, or field measurements—are combined with visual features to provide a more complete evaluation of both the causes and consequences of structural damage.

## Figures and Tables

**Table 1:** The architectural overview of the LFE block.

Layer Name	Type	Kernel Size	Activation
Input	Feature Map	-	-
Conv	Convolution	3 × 3	-
Conv	Convolution	3 × 3	-
BN	Batch Normalization	-	-
Connection	Residual Connection	-	-
Conv	Convolution	1 × 1	ReLU
BN	Batch Normalization	-	ReLU

**Table 2:** The impact of each component on the performance of the models.

Configuration	Accuracy	F1-Score	Notes
DETR (baseline)	91.05	0.89	Without ViT
ViT + DETR	93.11	0.90	With ViT (without LFE)
ViT + DETR	95.0	0.93	With ViT (with LFE)

**Table 3:** Quantitative performance comparison results.

Model	Accuracy (%)	Precision	Recall	F1-Score	mAP@0.5
DETR (baseline)	91.5	0.89	0.88	0.89	0.82
ViT-DETR	93.11	0.91	0.90	0.90	0.85
YOLOv5m	91.12	0.86	0.85	0.86	0.81
YOLOv6m	91.67	0.89	0.88	0.88	0.82
YOLOv7m	92.78	0.91	0.90	0.90	0.85
YOLOv8m	93.89	0.92	0.91	0.92	0.86
YOLOv9m	94.16	0.93	0.92	0.92	0.86
**Ours (** **ViT+** **LFE-DETR)**	**95.** **0**	**0.94**	**0.93**	**0.93**	**0.89**

**Table 4:** Below is a summary of quantitative comparisons, including key models from each category.

Model	Accuracy (%)	Precision	Recall	F1-Score	mAP@0.5	Category	Reference
Canny + Sobel [14]	72.3	0.65	0.60	0.62	–	Traditional	Jahanshahi et al.
Wavelet + IR [15]	76.4	0.69	0.65	0.67	–	Traditional	Saleh et al.
Shallow CNN [20]	84.1	0.81	0.79	0.80	0.74	CNN	Kim et al.
DeepLabV3+ [22]	88.9	0.87	0.85	0.86	0.78	CNN	Song et al.
YOLOv5m [27]	91.12	0.86	0.85	0.86	0.81	CNN (YOLO)	Lin et al.
YOLOv9m [27]	94.16	0.93	0.92	0.92	0.86	CNN (YOLO)	Lin et al.
DETR [25]	91.05	0.89	0.88	0.89	0.82	Transformer	Carion et al.
Adaln [9]	91.5	0.90	0.89	0.89	0.83	Transformer	Guo et al.
ViT-DETR [24]	93.11	0.91	0.90	0.90	0.85	Transformer	Shahin et al.
CrackViT [29]	93.8	0.92	0.91	0.91	0.86	Hybrid CNN + Transformer	Quan et al.
FCFNet [30]	93.9	0.92	0.91	0.91	0.86	Hybrid CNN + Transformer	Ma et al.
ETAFHrNet [31]	94.2	0.93	0.92	0.92	0.86	Hybrid CNN + Transformer	Tan et al.
**ViLFD** **(Ours)**	**95.0**	**0.94**	**0.93**	**0.93**	**0.89**	**Hybrid** **ViT** **+** **LFE**	This Work

## Data Availability

All used datasets are available online via open access.

## References

[1] Nicolini E. (2024). Urban Safety, Socio-Technical Solutions for Urban Infrastructure: Case Studies. Buildings.

[2] Wang M., Yin X. (2022). Construction and maintenance of urban underground infrastructure with digital technologies. Autom. Constr..

[3] Brunelli M., Ditta C.C., Postorino M.N. (2023). New infrastructures for Urban Air Mobility systems: A systematic review on vertiport location and capacity. J. Air Transp. Manag..

[4] Manivasakan H., Kalra R., O’Hern S., Fang Y., Xi Y., Zheng N. (2021). Infrastructure requirement for autonomous vehicle integration for future urban and suburban roads–Current practice and a case study of Melbourne, Australia. Transp. Res. Part A Policy Pract..

[5] Abdusalomov A., Umirzakova S., Kutlimuratov A., Mirzaev D., Dauletov A., Botirov T., Zakirova M., Mukhiddinov M., Cho Y.I. (2025). Lightweight UAV-Based System for Early Fire-Risk Identification in Wild Forests. Fire.

[6] Oh B.K., Park H.S. (2022). Urban safety network for long-term structural health monitoring of buildings using convolutional neural network. Autom. Constr..

[7] Oulahyane A., Kodad M., Bouazza A., Oulahyane K. Assessing the Impact of Deep Learning on Grey Urban Infrastructure Systems: A Comprehensive Review. Proceedings of the 2024 International Conference on Decision Aid Sciences and Applications (DASA).

[8] Rane N. (2023). Transformers in Intelligent Architecture, Engineering, and Construction (AEC) Industry: Applications, Challenges, and Future Scope. SSRN Electron. J..

[9] Guo Y., Wang C., Yu S.X., McKenna F., Law K.H. (2022). Adaln: A vision transformer for multidomain learning and predisaster building information extraction from images. J. Comput. Civ. Eng..

[10] Ashayeri M., Abbasabadi N. (2024). Unraveling energy justice in NYC urban buildings through social media sentiment analysis and transformer deep learning. Energy Build..

[11] Abdusalomov A., Umirzakova S., Shukhratovich M.B., Kakhorov A., Cho Y.-I. (2025). Breaking New Ground in Monocular Depth Estimation with Dynamic Iterative Refinement and Scale Consistency. Appl. Sci..

[12] Kosova F., Altay Ö., Ünver H.Ö. (2025). Structural health monitoring in aviation: A comprehensive review and future directions for machine learning. Nondestruct. Test. Eval..

[13] Mohebbifar M.R., Omarmeli K. (2020). Defect detection by combination of threshold and multistep watershed techniques. Russ. J. Nondestruct. Test..

[14] Jahanshahi M.R., Kelly J.S., Masri S.F., Sukhatme G.S. (2009). A survey and evaluation of promising approaches for automatic image-based defect detection of bridge structures. Struct. Infrastruct. Eng..

[15] Saleh A.K., Sakka Z., Almuhanna H. (2022). The application of two-dimensional continuous wavelet transform based on active infrared thermography for subsurface defect detection in concrete structures. Buildings.

[16] Lin Y.Z., Nie Z.H., Ma H.W. (2017). Structural damage detection with automatic feature-extraction through deep learning. Comput.-Aided Civ. Infrastruct. Eng..

[17] Zhu H., Wei G., Ma D., Yu X., Dong C. (2025). Research on the detection and identification method of internal cracks in semi-rigid base asphalt pavement based on three-dimensional ground penetrating radar. Measurement.

[18] Karimi N., Mishra M., Lourenço P.B. (2025). Automated surface crack detection in historical constructions with various materials using deep learning-based YOLO network. Int. J. Archit. Herit..

[19] Krishnan S.S.R., Karuppan M.N., Khadidos A.O., Khadidos A.O., Selvarajan S., Tandon S., Balusamy B. (2025). Comparative analysis of deep learning models for crack detection in buildings. Sci. Rep..

[20] Kim B., Yuvaraj N., Sri Preethaa K.R., Arun Pandian R. (2021). Surface crack detection using deep learning with shallow CNN architecture for enhanced computation. Neural Comput. Appl..

[21] Abbas Y.M., Alghamdi H. (2025). Semantic segmentation and deep CNN learning vision-based crack recognition system for concrete surfaces: Development and implementation. Signal Image Video Process..

[22] Song F., Wang D., Dai L., Yang X. Concrete bridge crack semantic segmentation method based on improved DeepLabV3+. Proceedings of the 2024 IEEE 13th Data Driven Control and Learning Systems Conference (DDCLS).

[23] Liu Y. (2025). DeepLabV3+ Based Mask R-CNN for Crack Detection and Segmentation in Concrete Structures. Int. J. Adv. Comput. Sci. Appl..

[24] Shahin M., Chen F.F., Maghanaki M., Hosseinzadeh A., Zand N., Khodadadi Koodiani H. (2024). Improving the concrete crack detection process via a hybrid visual transformer algorithm. Sensors.

[25] Carion N., Massa F., Synnaeve G., Usunier N., Kirillov A., Zagoruyko S. (2020). End-to-end object detection with transformers. European Conference on Computer Vision.

[26] Qingyi W., Bo C. (2024). A novel transfer learning model for the real-time concrete crack detection. Knowl.-Based Syst..

[27] Lin X., Meng Y., Sun L., Yang X., Leng C., Li Y., Niu Z., Gong W., Xiao X. (2025). Building Surface Defect Detection Based on Improved YOLOv8. Buildings.

[28] Yadav D.P., Sharma B., Chauhan S., Dhaou I.B. (2024). Bridging Convolutional Neural Networks and Transformers for Efficient Crack Detection in Concrete Building Structures. Sensors.

[29] Quan J., Ge B., Wang M. (2023). CrackViT: A unified CNN-transformer model for pixel-level crack extraction. Neural Comput. Appl..

[30] Ma M., Yang L., Liu Y., Yu H. (2024). A transformer-based network with feature complementary fusion for crack defect detection. IEEE Trans. Intell. Transp. Syst..

[31] Tan C., Liu J., Zhao Z., Liu R., Tan P., Yao A., Pan S., Dong J. (2025). ETAFHrNet: A Transformer-Based Multi-Scale Network for Asymmetric Pavement Crack Segmentation. Appl. Sci..

